# Supplementary material for: HDX reveals the conformational dynamics of DNA sequence specific VDR co-activator interactions
Source: Nat Commun. 2017 Oct 13;8:923. doi: 10.1038/s41467-017-00978-7 (PMC5640644; doi:10.1038/s41467-017-00978-7)
Supplement: Supplementary file 1 — Supplementary Information [file 41467_2017_978_MOESM1_ESM.pdf]

Supplementary Figure 1a

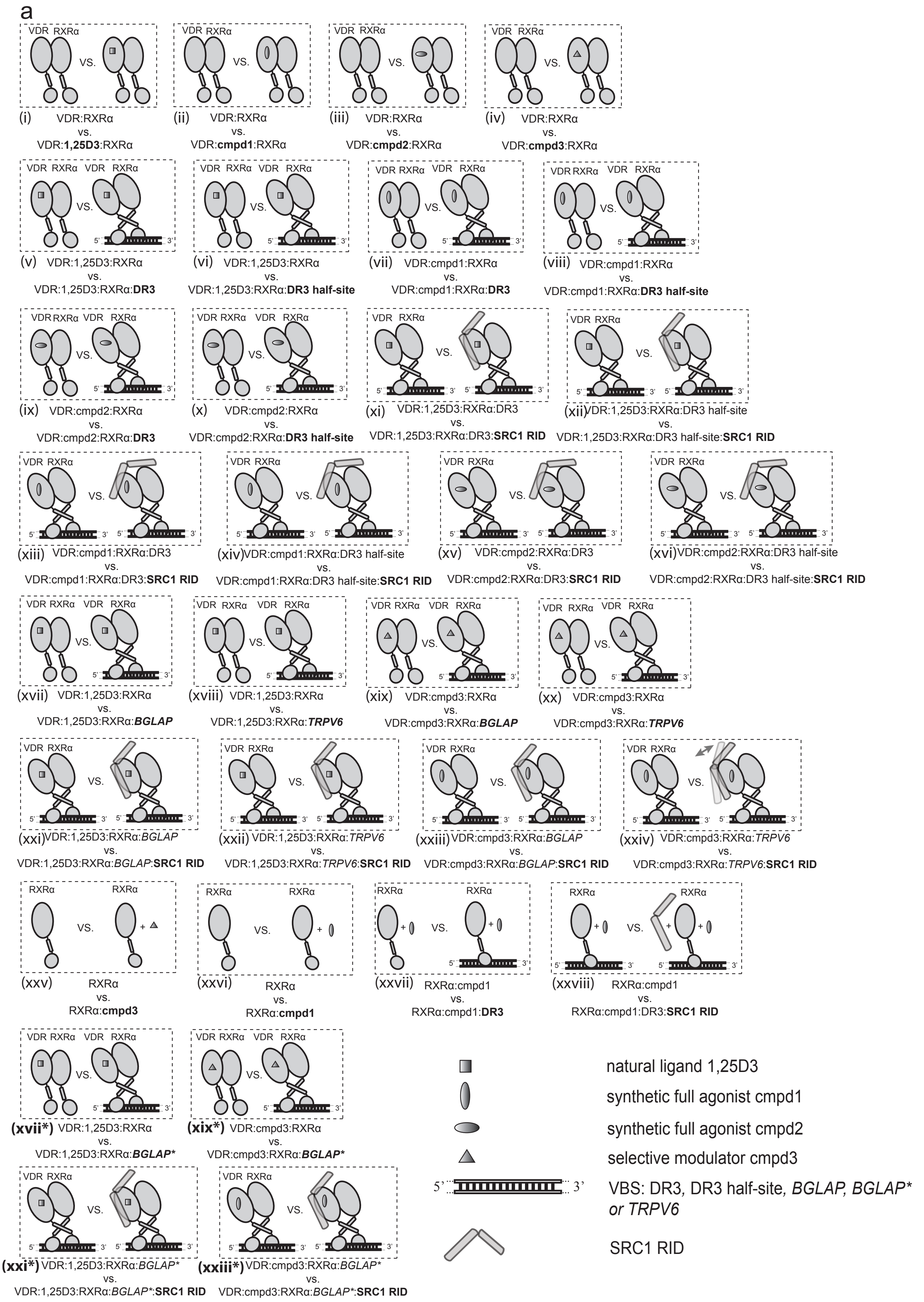

Peptide sequence	charge	start	end	structure	(i)	(ii)	(iii)	(iv)	(v)	(vi)	(vii)	(viii)	(ix)	(x)	(xi)	(xii)	(xiii)	(xiv)	(xv)	(xvi)	(xvii)	(xviii)	(xix)	(xx)	(xxi)	(xxii)	(xxiii)	(xxiv)	(xvii*)	(xix*)	(xxi*)	(xxiii*)
AMAASTSLPDPGDF	2	3	16		-1 (2)*	-3 (5)*	-2 (6)*	-2 (4)*	1 (3)*	-8 (4)	-8 (4)	-8 (6)	-4 (5)*	-5 (5)*	3 (3)*	0 (4)*	3 (2)*	4 (3)*	0 (4)*	-2 (4)*	0 (3)*	-1 (5)*	-1 (4)*	-2 (4)*	1 (4)*	3 (1)*	3 (3)*	3 (4)*	-2 (5)*	-1 (3)*	-1 (4)*	2 (4)*
MAASTSLPDPGDF	1	4	16	A/B domain	1 (6)*	2 (5)*	1 (6)*	-1 (4)*	-2 (5)*	-12 (4)	-11 (5)	-10 (5)	-5 (6)	-7 (6)	1 (2)*	-1 (4)*	1 (3)*	2 (3)*	1 (4)*	1 (4)*	-1 (4)*	-2 (5)*	-1 (3)*	-3 (5)*	-1 (6)*	3 (4)*	2 (4)*	2 (4)*	-3 (5)*	-2 (2)*	-1 (4)*	0 (3)*
MAASTSLPDPGDF	2	4	16		1 (4)*	1 (5)*	1 (5)*	0 (4)*	1 (5)*	-12 (3)	-11 (3)	-8 (4)	-6 (5)	-7 (5)	1 (3)*	0 (4)*	2 (3)*	3 (2)*	1 (3)*	1 (4)*	-2 (4)*	-4 (4)*	2 (4)*	0 (5)*	0 (4)*	2 (4)*	1 (4)*	3 (5)*	-1 (4)*	-2 (2)*	-3 (6)*	3 (3)*
DRNVPRICG	2	17	25		1 (2)*	2 (5)*	1 (5)*	0 (4)*	-13 (4)	-22 (6)	-30 (3)	-25 (6)	-21 (5)	-21 (5)	2 (4)*	1 (3)*	2 (3)*	-2 (3)*	N/A	1 (3)*	-9 (5)	-18 (4)	-9 (4)	-15 (4)	4 (5)*	2 (4)*	0 (4)*	2 (5)*	-14 (4)	-10 (3)	-1 (5)*	0 (4)*
DRNVPRICG	3	17	25		1 (3)*	2 (5)*	1 (5)*	-1 (4)*	-12 (4)	-21 (6)	-29 (4)	-25 (6)	-22 (5)	-22 (5)	N/A	0 (4)*	4 (3)*	0 (3)*	3 (5)*	3 (5)*	-11 (3)	-20 (3)	-9 (4)	-14 (4)	-1 (4)*	2 (4)*	-1 (4)*	3 (2)*	-14 (5)	-10 (4)	-2 (5)*	1 (3)*
VCGDRATGFHF	2	26	36		2 (4)*	2 (4)*	1 (4)*	-3 (4)*	-26 (3)	-22 (3)	-44 (3)	-28 (6)	-17 (4)	-23 (3)	1 (4)*	-2 (4)*	3 (5)*	2 (4)*	-1 (6)*	-4 (4)*	-29 (4)	-31 (4)	-20 (3)	-29 (7)	-2 (4)*	-4 (5)*	-1 (3)*	0 (4)*	-28 (5)	-15 (5)	1 (4)*	-2 (4)*
NAMTCEGCKG	2	37	46		1 (2)*	2 (4)*	-1 (3)*	-1 (3)*	-22 (3)	-26 (3)	-35 (5)	-28 (3)	-31 (3)	-25 (4)	2 (3)*	0 (2)*	3 (2)*	2 (3)*	N/A	-1 (3)*	-27 (6)	-27 (5)	-18 (6)	-22 (6)	0 (3)*	1 (6)*	2 (3)*	-1 (5)*	-23 (4)	-19 (3)	-1 (3)*	-2 (3)*
NAMTCEGCKGF	2	37	47		2 (3)*	3 (3)*	2 (3)*	1 (3)*	-26 (4)	-27 (5)	-38 (3)	-30 (3)	-36 (2)	-31 (3)	0 (3)*	-4 (3)*	-2 (3)*	-3 (2)*	-6 (4)	-2 (2)*	-26 (6)	-30 (5)	-18 (4)	-28 (4)	-3 (4)*	-1 (4)*	-4 (4)*	-1 (3)*	-22 (6)	-14 (4)	-5 (4)*	-4 (4)*
NAMTCEGCKGFF	2	37	48		1 (5)*	3 (3)*	2 (2)*	2 (3)*	-25 (3)	-26 (5)	-36 (3)	-29 (3)	-30 (2)	-25 (3)	1 (3)*	-1 (2)*	1 (2)*	0 (3)*	-3 (4)*	-3 (3)*	-25 (5)	-32 (4)	-19 (3)	-27 (4)	-4 (5)*	0 (4)*	-2 (4)*	-2 (5)*	-24 (4)	-16 (5)	-1 (5)*	0 (6)*
MTCEGCKGFF	2	39	48	DBD	4 (4)*	4 (3)*	3 (2)*	3 (3)*	-24 (3)	-27 (4)	-34 (3)	-28 (3)	-28 (2)	-24 (3)	0 (3)*	1 (2)*	4 (2)*	2 (2)*	2 (3)*	1 (2)*	-22 (5)	-28 (4)	-17 (3)	-22 (4)	2 (3)*	5 (4)*	0 (3)*	2 (3)*	-20 (4)	-13 (6)	-1 (4)*	4 (5)*
TCEGCKGFF	2	40	48		3 (3)*	4 (3)*	3 (3)*	2 (3)*	-25 (3)	-27 (4)	-33 (3)	-27 (3)	-27 (2)	-23 (3)	-1 (3)*	-2 (2)*	-1 (2)*	-1 (3)*	-4 (4)*	-4 (2)*	-22 (4)	-28 (5)	-17 (3)	-25 (3)	-4 (5)*	0 (3)*	-4 (4)*	-2 (4)*	-19 (4)	-13 (4)	-2 (3)*	-1 (5)*
FTCPFNGD	1	58	65		2 (4)*	3 (6)*	2 (5)*	-2 (4)*	-20 (4)	-28 (2)	-29 (4)	-25 (3)	-19 (5)	-18 (6)	N/A	-3 (4)*	2 (3)*	1 (4)*	-3 (5)*	-1 (4)*	-12 (4)	-25 (5)	-8 (5)	-16 (5)	-1 (3)*	-1 (6)*	-1 (3)*	-5 (5)*	-15 (5)	-8 (2)	-4 (3)*	-3 (4)*
FTCPFNGDC	1	58	66		2 (3)*	3 (5)*	-1 (5)*	0 (5)*	-18 (5)	-23 (3)	-33 (3)	-27 (5)	-24 (6)	-21 (5)	-1 (3)*	-2 (5)*	1 (3)*	-1 (3)*	2 (6)*	3 (4)*	-17 (4)	-25 (5)	-7 (3)	-14 (5)	-1 (3)*	-2 (4)*	0 (4)*	-1 (3)*	-15 (6)	-10 (2)	1 (2)*	0 (5)*
RITKDNRRHCQA	2	67	78		2 (4)*	2 (4)*	1 (4)*	2 (3)*	-15 (5)	-21 (4)	-24 (4)	-21 (3)	-11 (3)	-11 (5)	1 (5)*	1 (3)*	-1 (0)*	2 (2)*	1 (6)*	-1 (5)*	-13 (1)	-17 (4)	-7 (4)	-14 (3)	-1 (6)*	N/A	-2 (3)*	N/A	N/A	N/A	N/A	2 (6)*
KRCVDIGM	2	82	89		9 (5)	9 (3)	7 (2)	7 (3)	-34 (3)	-36 (4)	-41 (2)	-41 (4)	-38 (3)	-34 (4)	1 (1)*	2 (2)*	2 (1)*	-3 (2)*	1 (5)*	-2 (3)*	-23 (4)	-29 (3)	-19 (4)	-25 (5)	0 (4)*	0 (1)*	-3 (3)*	-2 (3)*	-27 (4)	-15 (4)	0 (2)*	-1 (3)*
IGMMKE	2	87	92		1 (4)*	3 (4)*	0 (2)*	-10 (5)	-31 (5)	-26 (4)	-38 (3)	-37 (5)	-40 (5)	-35 (6)	N/A	-3 (4)*	5 (3)*	1 (3)*	-1 (6)*	0 (6)*	-24 (3)	-35 (5)	-20 (5)	-29 (4)	0 (4)*	2 (3)*	-1 (4)*	0 (5)*	-31 (5)	-11 (4)	0 (4)*	0 (4)*
FILTDEE	1	93	99		0 (2)*	1 (5)*	2 (5)*	-19 (5)	-31 (5)	-23 (3)	-46 (4)	-26 (3)	-35 (5)	-20 (3)	1 (5)*	-1 (2)*	2 (6)*	2 (3)*	0 (5)*	-5 (5)	-31 (3)	-39 (5)	-24 (5)	-29 (5)	-4 (4)*	-1 (6)*	-2 (4)*	4 (4)*	-42 (4)	-14 (4)	0 (4)*	1 (4)*
DEEVQRKREMIL	3	97	108	Hinge	1 (5)*	1 (5)*	0 (4)*	-15 (5)	-49 (4)	-45 (5)	-70 (4)	-49 (5)	-54 (5)	-35 (4)	2 (2)*	-2 (4)*	-3 (2)*	0 (4)*	N/A	N/A	-52 (4)	-58 (3)	-48 (5)	-51 (5)	-2 (5)*	-5 (4)	-1 (3)*	0 (2)*	-45 (4)	-35 (5)	-1 (4)*	-2 (5)*
EVQRKREMIL	2	99	108		-1 (4)*	1 (5)*	1 (5)*	-22 (6)	-47 (4)	-32 (5)	-67 (5)	-46 (3)	-54 (6)	-35 (4)	2 (2)*	-3 (3)*	-2 (4)*	-2 (6)*	-5 (8)	0 (3)*	-50 (5)	-59 (5)	-40 (6)	-44 (5)	-2 (3)*	1 (4)*	1 (5)*	0 (4)*	-43 (3)	-32 (4)	-2 (4)*	-2 (3)*
KRKEEEALKDSLRPKLSEEQQRIIA	4	109	133	Hinge, H1	-1 (5)*	0 (3)*	0 (3)*	-4 (4)*	-8 (4)	-14 (4)	-14 (3)	-15 (5)	-13 (3)	-10 (3)	0 (2)*	0 (3)*	3 (3)*	2 (3)*	-1 (3)*	0 (6)*	3 (0)*	1 (3)*	-5 (3)	-13 (5)	3 (6)*	N/A	4 (2)*	0 (3)*	-15 (6)	-2 (3)*	-2 (4)*	-2 (3)*
SEEQQRIIAIL	2	125	135	Hinge, H1	0 (2)*	0 (2)*	0 (2)*	0 (1)*	-4 (1)*	-7 (2)	-6 (1)	-6 (2)	-3 (2)*	-4 (2)*	4 (3)*	-3 (1)*	-4 (1)*	0 (2)*	0 (3)*	0 (3)*	-2 (2)*	-2 (3)*	-1 (2)*	-3 (2)*	-3 (2)*	-4 (3)*	-3 (2)*	-2 (3)*	-2 (2)*	-1 (2)*	4 (3)*	2 (4)*
ILLDAHHKTYDPTYSDF	3	134	150		-28 (2)	-10 (3)	-19 (2)	-16 (2)	-1 (2)*	-3 (2)*	-8 (3)	-7 (2)	-4 (2)*	-5 (2)*	-7 (4)	-6 (2)	4 (2)*	5 (3)*	-5 (3)	-5 (3)	-2 (3)*	-3 (3)*	-5 (2)	-7 (4)	1 (1)*	0 (4)*	-3 (4)*	-1 (3)*	-1 (3)*	-7 (4)	-1 (2)*	4 (5)*
LDAHHKTYDPTYSDF	4	136	150	H1	-30 (3)	-12 (3)	-22 (3)	-18 (2)	-1 (2)*	-4 (3)*	-11 (5)	-9 (3)	-7 (3)	-7 (2)	N/A	-8 (2)	1 (4)*	-1 (3)*	-6 (3)	-6 (3)	-3 (3)*	-3 (4)*	-6 (3)	-7 (3)	0 (3)*	-1 (4)*	-6 (3)	-4 (4)*	-1 (3)*	-7 (5)	-3 (3)*	0 (9)*
DAHHKTYDPTYSDF	3	137	150		-32 (4)	-12 (4)	-24 (3)	-20 (3)	-2 (3)*	-6 (3)	-13 (3)	-12 (3)	-7 (3)	-8 (3)	-11 (4)	-7 (4)	-1 (2)*	0 (3)*	-6 (4)	-6 (4)	-3 (4)*	-2 (2)*	-7 (3)	-8 (3)	0 (4)*	-1 (6)*	0 (3)*	-1 (4)*	-1 (4)*	N/A	-3 (3)*	0 (4)*
HCITSSDM	2	189	196		0 (3)*	1 (6)*	2 (6)*	-2 (4)*	N/A	-27 (4)	-32 (3)	-25 (2)	-19 (3)	-21 (6)	N/A	4 (0)*	4 (4)*	0 (3)*	0 (7)*	-2 (3)*	-2 (4)*	-19 (5)	0 (4)*	-11 (5)	2 (3)*	3 (6)*	2 (2)*	2 (3)*	-4 (6)*	-4 (3)*	-2 (1)*	3 (4)*
MDSSSFSNLDL	2	197	207	loop	0 (1)*	-1 (2)*	0 (1)*	-1 (5)*	-1 (3)*	-16 (4)	-9 (2)	-7 (3)	N/A	-6 (4)	N/A	-2 (1)*	1 (3)*	-5 (2)*	-4 (1)*	3 (3)*	0 (2)*	0 (5)*	0 (3)*	-6 (2)	-2 (1)*	5 (3)*	1 (3)*	1 (2)*	-3 (4)*	-3 (3)*	0 (4)*	5 (3)*
DLSEEDSDDPSVT	1	206	218		2 (3)*	2 (5)*	4 (6)*	-3 (5)*	-4 (6)*	-17 (6)	-13 (6)	-9 (4)	-10 (5)	-11 (5)	1 (4)*	0 (4)*	1 (4)*	2 (2)*	2 (3)*	2 (4)*	-2 (4)*	-3 (3)*	-3 (4)*	-5 (5)	0 (2)*	3 (4)*	1 (3)*	3 (4)*	-4 (5)*	-2 (3)*	-1 (6)*	1 (4)*
DLSEEDSDDPSVT	2	206	218		0 (4)*	2 (5)*	4 (5)*	-3 (4)*	-4 (6)*	-17 (5)	-12 (6)	-9 (4)	-10 (5)	-11 (4)	N/A	0 (4)*	2 (3)*	3 (3)*	1 (3)*	1 (3)*	-1 (4)*	-1 (3)*	-1 (3)*	-7 (5)	2 (2)*	3 (4)*	0 (4)*	4 (3)*	-2 (4)*	-2 (3)*	0 (4)*	3 (4)*
SQLSMLPHLADL	2	222	233	H2, H3	-46 (5)	-47 (4)	-41 (4)	-35 (4)	-1 (4)*	-4 (3)*	-7 (3)	-7 (2)	-7 (2)	-8 (2)	-18 (3)	-9 (3)	0 (3)*	0 (2)*	-7 (3)	-6 (3)	-2 (4)*	-1 (3)*	-8 (4)	-10 (5)	-2 (3)*	0 (4)*	-7 (5)	-2 (5)*	-1 (4)*	-6 (3)	-1 (3)*	-10 (4)
SMLPHLADL	2	225	233		-60 (6)	-63 (4)	-54 (4)	-43 (4)	0 (3)*	-1 (3)*	-6 (2)	-6 (2)	-7 (2)	-8 (2)	-15 (5)	-10 (5)	-3 (1)*	-3 (1)*	-9 (3)	-9 (2)	-2 (3)*	-1 (3)*	-10 (5)	-10 (4)	-2 (1)*	-1 (3)*	-9 (6)	-6 (3)	-1 (1)*	-6 (2)	-1 (1)*	-10 (4)
VSYSIQKVIGF	2	234	244	H3	-41 (4)	-52 (5)	-51 (5)	-36 (4)	0 (1)*	0 (1)*	-3 (4)*	-2 (1)*	-2 (1)*	-2 (1)*	-12 (3)	-4 (3)*	3 (1)*	-2 (18)*	-1 (1)*	-1 (1)*	1 (6)*	-1 (3)*	-5 (2)	-8 (6)	0 (4)*	-1 (1)*	-2 (4)*	0 (5)*	-1 (2)*	-3 (5)*	3 (2)*	0 (4)*
SIQKVIGF	2	237	244		-30 (4)	-40 (4)	-39 (4)	-24 (3)	-1 (1)*	-1 (1)*	-2 (1)*	-3 (1)*	-3 (1)*	-3 (1)*	-7 (3)	-2 (3)*	-1 (1)*	-1 (1)*	-2 (1)*	-2 (1)*	-1 (1)*	-2 (2)*	-4 (3)*	-3 (1)*	2 (6)*	1 (3)*	-1 (2)*	0 (2)*	-1 (1)*	-1 (3)*	1 (1)*	-2 (3)*
FAKMIPGFRDLTSEDQ	3	244	259		1 (4)*	1 (2)*	0 (2)*	-1 (1)*	-2 (2)*	-7 (3)	-7 (3)	-5 (2)	-4 (2)*	-5 (2)*	0 (4)*	-2 (1)*	1 (3)*	0 (2)*	-2 (2)*	-2 (2)*	-2 (4)*	-4 (2)*	-1 (2)*	-3 (2)*	-2 (3)*	0 (5)*	0 (4)*	-1 (2)*	-1 (3)*	-2 (3)*	-3 (2)*	0 (5)*
AKMIPGFRDLTSED	2	245	258	H3, H4	0 (3)*	1 (2)*	0 (2)*	-2 (1)*	-3 (2)*	-9 (3)	-8 (4)	-6 (3)	-6 (3)	-6 (3)	4 (4)*	-1 (3)*	2 (3)*	0 (3)*	-1 (2)*	-1 (2)*	-1 (4)*	-4 (4)*	-2 (2)*	-4 (3)*	1 (2)*	0 (9)*	1 (4)*	0 (4)*	-1 (4)*	-2 (3)*	0 (3)*	2 (6)*
AKMIPGFRDLTSEDQ	2	245	259		1 (3)*	1 (2)*	0 (2)*	-2 (1)*	-3 (2)*	-9 (3)	-6 (3)	-6 (2)	-6 (2)	-6 (2)	0 (4)*	-2 (2)*	-1 (2)*	-1 (3)*	-3 (2)*	-3 (2)*	-2 (4)*	-3 (4)*	-1 (2)*	-4 (3)*	-5 (2)	-2 (4)*	1 (4)*	0 (4)*	-1 (4)*	-1 (3)*	-3 (3)*	0 (6)*
LKSSAIEV	2	263	270	H4, H5	N/A	-14 (2)	-14 (2)	-6 (2)	0 (1)*	-1 (1)*	-1 (1)*	-1 (1)*	-1 (1)*	-2 (1)*	-1 (2)*	N/A	N/A	N/A	0 (1)*	0 (1)*	N/A	1 (0)*	N/A	-1 (1)*	N/A	N/A	N/A	1 (1)*	N/A	-1 (2)*	1 (0)*	N/A
RSNESFTMDDMS	2	274	285	H5, S1	-39 (5)	-34 (4)	-37 (4)	-27 (3)	-4 (3)*	-10 (4)	-10 (4)	-10 (5)	-7 (2)	-12 (3)	-2 (4)*	-7 (3)	0 (2)*	-1 (3)*	4 (4)*	-3 (3)*	-2 (3)*	-3 (3)*	-6 (3)	-9 (3)	0 (3)*	-1 (5)*	-7 (5)	-2 (4)*	-2 (4)*	-7 (4)	-2 (3)*	-2 (4)*
MSWTCGNQDYKYRVSD	3	284	299	S2, H6	-37 (3)	-34 (6)	-34 (3)	-31 (4)	N/A	-25 (4)	-19 (3)	-18 (6)	-15 (4)	-17 (5)	-1 (2)*	-4 (4)*	1 (3)*	2 (3)*	0 (5)*	N/A	-1 (3)*	-2 (4)*	-8 (3)	-15 (5)	0 (5)*	1 (6)*	-2 (4)*	N/A	-3 (6)*	-6 (4)	-1 (4)*	0 (2)*
VTKAGHSLE	2	300	308	H6, H7	-32 (3)	-25 (8)	N/A	-19 (4)	-3 (1)*	-12 (3)	-18 (3)	-17 (4)	-16 (3)	-18 (3)	-2 (4)*	-5 (4)*	4 (4)*	1 (2)*	-2 (6)*	-3 (3)*	-3 (4)*	-4 (3)*	-7 (4)	-11 (3)	-2 (3)*	-1 (5)*	-4 (4)*	1 (6)*	-3 (4)*	-6 (3)	-2 (4)*	-1 (4)*
LIEPLIKF	2	309	316	H7	-32 (4)	-41 (4)	-40 (4)	-26 (3)	0 (1)*	0 (1)*	-3 (1)*	-3 (1)*	-4 (1)*	-4 (1)*	-5 (4)	-5 (3)	-1 (1)*	-1 (1)*	-1 (1)*	-1 (1)*	-2 (1)*	-1 (2)*	-5 (2)	-4 (2)*	-1 (1)*	-1 (1)*	-2 (3)*	-1 (3)*	-1 (1)*	-2 (3)*	0 (1)*	-2 (3)*
IEPLIKF	2	310	316		-29 (4)	-39 (5)	-39 (5)	-25 (4)	0 (1)*	-1 (1)*	-3 (1)*	-3 (1)*	-3 (1)*	-3 (1)*	1 (2)*	-3 (2)*	0 (3)*	2 (3)*	4 (4)*	3 (4)*	-2 (2)*	-2 (3)*	-5 (2)*	-3 (2)*	-1 (1)*	-1 (1)*	4 (4)*	-1 (2)*	-2 (1)*	-2 (3)*	-2 (1)*	4 (3)*
EEHVLLM	2	328	334	H8	-1 (3)*	N/A	N/A	1 (2)*	4 (4)*	0 (3)*	0 (2)*	N/A	N/A	N/A	-1 (2)*	-1 (3)*	1 (3)*	2 (3)*	-1 (2)*	-3 (4)*	-2 (2)*	1 (4)*	-2 (1)*	-2 (3)*	-1 (2)*	N/A	-1 (4)*	1 (2)*	0 (4)*	-1 (1)*	-1 (1)*	N/A
IVSPDRPGVQD	2	338	348	H8, H9	-6 (3)	-3 (3)*	-6 (3)	-2 (2)*	-5 (3)*	-10 (2)	-19 (3)	-18 (3)	-14 (3)	-15 (3)	1 (3)*	2 (3)*	-3 (1)*	-4 (2)*	N/A	N/A	-3 (3)*	-2 (2)*	-5 (1)*	-6 (3)	0 (2)*	1 (3)*	1 (3)*	2 (2)*	-3 (3)*	-3 (4)*	-1 (2)*	0 (3)*
IEAIQDRLSNTLQT	2	352	365		0 (2)*	-1 (2)*	-2 (1)*	0 (1)*	0 (1)*	-1 (3)*	-1 (2)*	-1 (2)*	0 (1)*	-1 (1)*	0 (1)*	0 (2)*	-1 (2)*	0 (2)*	-2 (1)*	-1 (2)*	0 (1)*	0 (2)*	-1 (1)*	-1 (2)*	1 (2)*	-1 (1)*	-1 (1)*	-1 (4)*	-1 (1)*	-1 (1)*	-2 (1)*	0 (2)*
IEAIQDRLSNTLQTY	2	352	366	H9	0 (2)*	0 (2)*	-1 (1)*	0 (1)*	-1 (1)*	-1 (1)*	0 (2)*	0 (2)*	0 (1)*	0 (1)*	-1 (2)*	-1 (1)*	0 (2)*	-2 (2)*	-1 (1)*	-1 (2)*	-1 (1)*	-1 (1)*	0 (1)*	0 (1)*	2 (1)*	0 (6)*	0 (3)*	2 (4)*	-1 (1)*	0 (1)*	-1 (2)*	-2 (2)*
IQDRLSNTLQT	2	355	365		0 (2)*	-1 (2)*	0 (6)*	0 (1)*	-1 (1)*	-2 (2)*	-2 (2)*	-1 (3)*	-1 (4)*	-1 (4)*	1 (2)*	5 (3)*	-1 (2)*	-1 (1)*	0 (2)*	0 (3)*	-2 (2)*	-1 (2)*	0 (3)*	0 (2)*	0 (1)*	2 (1)*	0 (1)*	1 (1)*	0 (2)*	-1 (2)*	3 (1)*	1 (2)*
YIRCRHPPPGSHL	4	366	378	H9, H10	-1 (3)*	0 (2)*	0 (3)*	-2 (3)*	-3 (3)*	-13 (3)	-19 (2)	-17 (5)	-13 (4)	-14 (4)	1 (5)*	0 (3)*	0 (3)*	4 (2)*	2 (3)*	2 (3)*	-1 (4)*	-4 (3)*	-2 (2)*	-7 (3)	2 (5)*	2 (4)*	3 (4)*	0 (4)*	-3 (4)*	0 (5)*	0 (4)*	4 (4)*
YIRCRHPPPGSHLLYA	4	366	381		0 (3)*	-1 (3)*	-3 (3)*	-3 (4)*	-1 (3)*	1 (4)*	-10 (3)	-8 (3)	-4 (2)*	-5 (3)*	1 (4)*	1 (3)*	1 (3)*	1 (2)*	-2 (3)*	-2 (2)*	-3 (4)*	-3 (2)*	-2 (4)*	-5 (3)	-2 (4)*	-3 (5)*	1 (5)*	1 (3)*	-2 (4)*	-3 (4)*	0 (3)*	2 (2)*
IQKLAD	2	384	389	H10	1 (3)*	1 (2)*	-1 (2)*	-1 (1)*	-2 (1)*	-3 (1)*	-6 (1)	-6 (2)	-3 (1)*	-4 (1)*	0 (1)*	0 (1)*	0 (1)*	0 (1)*	-1 (1)*	-1 (1)*	0 (2)*	-2 (3)*	-2 (2)*	-2 (1)*	0 (1)*	0 (1)*	-1 (1)*	0 (1)*	-2 (1)*	-1 (2)*	1 (1)*	0 (2)*
LRSLNEEHSKQYRC	4	390	403	H11	-35 (5)	-47 (4)	-43 (3)	-28 (3)	-3 (4)*	-7 (4)	-9 (3)	-9 (2)	-9 (2)	-9 (2)	-13 (4)	-9 (3)	1 (2)*	1 (2)*	-4 (2)*	-4 (2)*	-2 (3)*	-3 (3)*	-11 (5)	-11 (3)	0 (3)*	4 (5)*	-5 (5)	-3 (4)*	-2 (3)*	-1 (5)*	0 (3)*	-1 (4)*
RSLNEEHSKQYRC	4	391	403		-37 (5)	-51 (4)	-45 (3)	-27 (4)	-2 (4)*	-7 (4)	-10 (2)	-8 (3)	-9 (2)	-9 (1)	-14 (4)	-10 (3)	1 (1)*	0 (2)*	-3 (4)*	-5 (2)	-3 (3)*	-1 (3)*	-6 (4)	-11 (3)	3 (2)*	-3 (5)*	-9 (4)	2 (1)*	-3 (4)*	2 (3)*	3 (3)*	-3 (4)*
YRCLSFQPECS	2	401	411	Loop	-24 (5)	-25 (4)	-21 (3)	-20 (3)	-2 (4)*	-13 (3)	-15 (3)	-14 (5)	-13 (5)	-13 (4)	-10 (4)	-11 (4)	1 (3)*	-2 (2)*	-14 (4)	-14 (4)	0 (4)*	-1 (2)*	-7 (4)	-9 (5)	-9 (4)	3 (5)*	-9 (5)	-1 (4)*	0 (3)*	-6 (3)	-2 (5)*	-14 (5)
LSFQPECS	1	404	411		-14 (6)	-14 (5)	-15 (5)	-18 (5)	-4 (2)*	-10 (4)	-21 (3)	-18 (4)	-16 (5)	-16 (5)	-14 (4)	-6 (4)	0 (4)*	-4 (4)*	-16 (7)	-15 (6)	-3 (3)*	-2 (3)*	-8 (4)	-10 (3)	-12 (5)	-1 (4)*	-11 (5)	-1 (3)*	0 (3)*	-7 (4)	-3 (3)*	-8 (5)
SMKLTPLVL	2	411	419	H12	-36 (3)	-47 (5)	-42 (5)	-36 (4)	-1 (2)*	-11 (2)	-12 (4)	-10 (5)	-8 (4)	-9 (5)	-21 (6)	-16 (4)	-11 (3)	-12 (3)	-22 (5)	-21 (5)	0 (4)*	1 (4)*	-2 (5)*	-13 (5)	-19 (5)	-19 (5)	-21 (6)	-9 (4)	-1 (5)*	-2 (4)*	-21 (5)	-18 (5)
MKLTPLVL	1	412	419		-37 (4)	-52 (6)	-45 (5)	-37 (4)	-2 (4)*	-9 (4)	-12 (4)	-9 (5)	-8 (4)	-9 (5)	-22 (5)	-16 (4)	-11 (4)	-12 (3)	-21 (5)	-21 (4)	-1 (4)*	-1 (5)*	-1 (5)*	-13 (4)	-19 (5)	-17 (5)	-21 (7)	-9 (5)	-1 (5)*	-1 (3)*	-20 (5)	-19 (5)

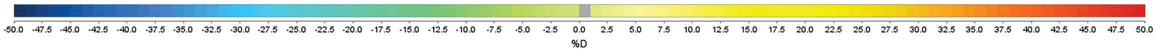

Supplementary Figure 1b

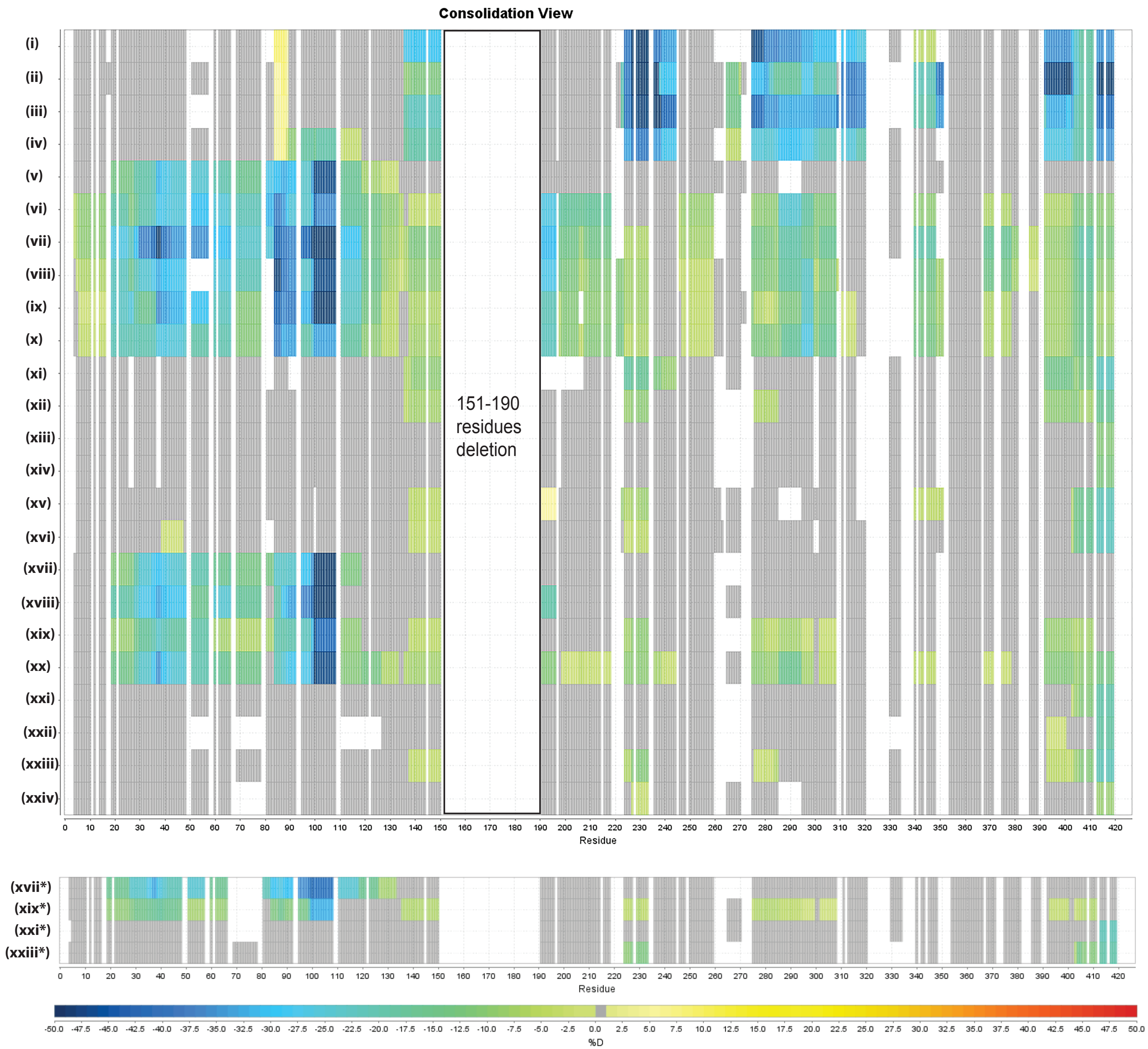

Supplementary Figure 1c

Peptide sequence	charge	start	end	structure	(i)	(ii)	(iii)	(iv)	(v)	(vi)	(vii)	(viii)	(ix)	(x)	(xi)	(xii)	(xiii)	(xiv)	(xv)	(xvi)	(xvii)	(xviii)	(xix)	(xx)	(xxi)	(xxii)	(xxiii)	(xxiv)	(xxv)	(xxvi)	(xxvii)	(xxviii)
DTKHFLPLDF	3	2	11	A/B domain	0 (7)*	1 (5)*	1 (5)*	-1 (4)*	2 (4)*	1 (5)*	4 (3)*	4 (6)*	7 (5)	6 (5)	0 (3)*	0 (3)*	-2 (3)*	-1 (5)*	-4 (4)*	-4 (2)*	1 (4)*	-2 (4)*	1 (3)*	-1 (5)*	0 (5)*	-1 (3)*	4 (3)*	0 (4)*	3 (5)*	-1 (4)*	-2 (3)*	0 (4)*
DTKHFLPLDF	2	2	11		2 (4)*	1 (4)*	2 (5)*	N/A	1 (4)*	0 (5)*	2 (4)*	3 (6)*	6 (5)	5 (5)	0 (3)*	3 (7)*	4 (4)*	0 (5)*	-3 (4)*	-3 (2)*	1 (5)*	-1 (4)*	1 (3)*	-1 (4)*	1 (4)*	2 (2)*	5 (3)*	4 (5)*	3 (5)*	-1 (4)*	-2 (3)*	0 (4)*
STQVNSSLTSPTGRGSMAAPSLHPSLGPGIGSPGQLHSPIST	4	12	53		0 (6)*	2 (5)*	1 (5)*	0 (5)*	3 (5)*	2 (5)*	2 (5)*	0 (6)*	2 (6)*	0 (6)*	5 (5)	0 (2)*	0 (3)*	-3 (5)*	-3 (5)*	-4 (1)*	2 (5)*	2 (6)*	1 (4)*	-1 (5)*	-1 (5)*	-2 (2)*	1 (4)*	-1 (3)*	3 (5)*	-1 (5)*	0 (4)*	0 (4)*
PSLHPSLGPGIGSPGQLHSPIST	3	31	53		0 (5)*	2 (5)*	1 (5)*	0 (5)*	1 (5)*	0 (6)*	-2 (5)*	-2 (6)*	-3 (5)*	-4 (5)*	3 (4)*	0 (4)*	2 (4)*	0 (5)*	-1 (4)*	-1 (1)*	-1 (5)*	0 (6)*	1 (3)*	-1 (5)*	-5 (5)*	0 (4)*	4 (4)*	1 (5)*	3 (5)*	0 (3)*	0 (4)*	1 (2)*
LSSPINGMGPPFSVISSPMGPHSMSVPTTPTL	3	54	85		2 (6)*	1 (6)*	0 (6)*	1 (5)*	3 (5)*	1 (6)*	2 (6)*	0 (7)*	1 (6)*	0 (6)*	N/A	0 (2)*	-4 (5)*	-4 (6)*	N/A	N/A	-1 (6)*	2 (0)*	1 (4)*	-2 (0)*	0 (3)*	2 (3)*	0 (4)*	N/A	3 (5)*	-3 (0)*	-1 (4)*	1 (5)*
FSTGSPQLSSPMNPVSSSE	2	87	105		-3 (0)*	0 (3)*	2 (3)*	1 (5)*	1 (5)*	0 (4)*	-4 (4)*	-5 (4)*	N/A	-5 (5)*	2 (6)*	2 (3)*	1 (4)*	2 (5)*	2 (4)*	2 (2)*	0 (5)*	0 (6)*	1 (4)*	-5 (0)*	3 (5)*	4 (4)*	4 (0)*	2 (5)*	4 (5)*	-1 (5)*	-2 (4)*	3 (5)*
DIKPPLGLNGVL	2	106	117		2 (3)*	1 (6)*	1 (6)*	-1 (6)*	4 (6)*	2 (7)*	-2 (6)*	-3 (6)*	-1 (6)*	-2 (6)*	1 (3)*	0 (4)*	0 (5)*	0 (5)*	-2 (5)*	-2 (2)*	1 (5)*	0 (6)*	1 (4)*	2 (4)*	0 (6)*	0 (4)*	2 (5)*	1 (4)*	3 (5)*	-1 (4)*	0 (3)*	1 (5)*
DIKPPLGLNGVLKVPAHPSGNMAS	3	106	129		2 (6)*	1 (5)*	1 (6)*	-1 (5)*	2 (5)*	1 (5)*	-2 (5)*	-3 (4)*	-2 (5)*	-2 (5)*	1 (3)*	1 (3)*	-2 (4)*	-2 (5)*	-2 (4)*	-3 (2)*	0 (5)*	0 (5)*	1 (3)*	-1 (5)*	-3 (4)*	-2 (3)*	-1 (4)*	-4 (2)*	3 (5)*	-2 (6)*	-2 (3)*	1 (2)*
GVLKVPAHPSGNMAS	3	115	129		3 (6)*	1 (6)*	1 (6)*	-1 (5)*	2 (5)*	0 (5)*	-1 (6)*	-3 (6)*	-5 (6)*	-5 (6)*	2 (0)*	1 (3)*	-2 (4)*	-2 (5)*	-1 (4)*	-1 (1)*	0 (5)*	0 (5)*	1 (3)*	-2 (4)*	2 (4)*	2 (2)*	5 (4)	1 (5)*	3 (5)*	-2 (5)*	-3 (4)*	1 (4)*
KVPAHPSGNMAS	3	118	129		2 (3)*	1 (2)*	2 (6)*	0 (6)*	-1 (4)*	-1 (6)*	-2 (5)*	-2 (7)*	-6 (2)	-7 (5)	3 (0)*	-1 (4)*	2 (6)*	1 (6)*	-3 (3)*	-5 (2)*	1 (7)*	0 (4)*	1 (5)*	-1 (3)*	0 (6)*	-3 (4)*	N/A	-1 (5)*	3 (5)*	0 (4)*	-2 (4)*	3 (2)*
FTKHICA	2	130	136	DBD	1 (4)*	1 (3)*	0 (3)*	0 (3)*	0 (3)*	-2 (4)*	-3 (3)*	-4 (4)*	-3 (3)*	-3 (3)*	2 (5)*	1 (2)*	0 (3)*	0 (3)*	-1 (2)*	-1 (1)*	-1 (3)*	-1 (3)*	-1 (3)*	-2 (3)*	0 (3)*	0 (2)*	1 (2)*	-1 (3)*	2 (4)*	0 (4)*	-1 (3)*	0 (4)*
ICGDRSSGKHYGVYSCEGCKGF	4	137	158		0 (3)*	1 (1)*	0 (2)*	-1 (2)*	-13 (3)	-10 (4)	-14 (2)	-10 (3)	-14 (2)	-10 (2)	0 (2)*	-2 (3)*	-3 (2)*	-2 (3)*	-3 (2)*	-3 (2)*	-12 (2)	-11 (3)	-12 (3)	-13 (3)	1 (3)*	-1 (5)*	N/A	-5 (3)*	3 (3)*	0 (4)*	-11 (4)	-3 (3)*
ICGDRSSGKHYGVYSCE	3	137	153		-1 (5)*	0 (3)*	-1 (2)*	-1 (2)*	-13 (3)	-11 (4)	-18 (4)	-13 (4)	-18 (2)	-14 (2)	1 (4)*	-2 (3)*	-2 (2)*	-5 (3)*	-2 (2)*	-3 (2)*	-13 (3)	-17 (4)	-13 (3)	-14 (4)	0 (2)*	2 (3)*	-1 (3)*	-2 (4)*	2 (4)*	0 (7)*	-13 (3)	0 (2)*
SCEGCKGFF	2	151	159		1 (4)*	1 (3)*	0 (2)*	N/A	-8 (2)	-7 (4)	-12 (3)	-9 (3)	-11 (2)	-8 (2)	0 (2)*	-2 (2)*	-1 (2)*	-4 (5)*	-2 (2)*	-2 (3)*	-8 (3)	-7 (4)	-10 (3)	-6 (3)	0 (3)*	-1 (2)*	-3 (3)*	-2 (4)*	2 (3)*	0 (4)*	-6 (3)	-2 (5)*
FFKRTVRKDLT	3	158	168		-1 (4)*	0 (4)*	-1 (5)*	0 (7)*	0 (3)*	-2 (5)*	-3 (5)*	-5 (5)*	-2 (4)*	-1 (2)*	0 (5)*	-1 (3)*	-4 (5)*	-1 (5)*	-3 (4)*	-3 (6)*	-5 (2)	N/A	-1 (2)*	-4 (3)*	-2 (3)*	-3 (4)*	0 (4)*	N/A	2 (3)*	1 (2)*	-1 (3)*	-1 (4)*
YTCRDNKDCL	2	169	178		-1 (3)*	-3 (0)*	0 (4)*	-2 (5)*	-12 (3)	-9 (4)	-19 (4)	-15 (4)	-19 (4)	-13 (2)	0 (5)*	1 (3)*	-1 (3)*	1 (4)*	1 (2)*	-2 (2)*	-10 (3)	-17 (4)	-13 (4)	-9 (3)	1 (2)*	5 (3)*	1 (3)*	-1 (4)*	0 (7)*	0 (3)*	-8 (4)	0 (4)*
CRYQKCLA	3	190	197		0 (3)*	0 (2)*	-1 (1)*	0 (2)*	-2 (1)*	-2 (2)*	-3 (2)*	-2 (2)*	-2 (1)*	-1 (1)*	5 (1)	-2 (1)*	-2 (1)*	-2 (2)*	-2 (1)*	-3 (1)*	-1 (1)*	-2 (1)*	-2 (3)*	-2 (1)*	-2 (1)*	-2 (1)*	-2 (1)*	-2 (3)*	1 (3)*	0 (3)*	-1 (1)*	-2 (3)*
VESTSSANEDMPVERILE	2	220	237	Hinge, H1	-2 (1)*	0 (3)*	-1 (5)*	2 (3)*	-3 (5)*	-5 (4)	-12 (5)	-10 (7)	-21 (3)	-15 (6)	0 (2)*	4 (4)*	-1 (2)*	-2 (4)*	N/A	4 (2)*	0 (3)*	-2 (3)*	0 (0)*	0 (4)*	0 (2)*	-1 (3)*	2 (3)*	-4 (4)*	3 (3)*	0 (4)*	-1 (3)*	2 (2)*
LAVEPKTET	2	240	248	H1, H2	1 (3)*	1 (5)*	1 (2)*	2 (4)*	0 (5)*	-1 (5)*	-7 (3)	-6 (4)	-8 (4)	-8 (5)	0 (3)*	3 (4)*	-2 (5)*	-1 (5)*	-1 (5)*	0 (3)*	0 (5)*	0 (7)*	-1 (5)*	-2 (5)*	5 (4)	2 (6)*	6 (4)	3 (6)*	5 (6)	-2 (4)*	-3 (5)*	2 (4)*
AVEPKTETYVE	2	241	251		1 (5)*	2 (6)*	-2 (3)*	0 (5)*	1 (5)*	0 (5)*	-6 (3)	-10 (6)	-8 (6)	-8 (6)	0 (4)*	3 (4)*	-1 (4)*	0 (4)*	3 (4)*	2 (2)*	0 (4)*	0 (6)*	-1 (5)*	-3 (6)*	3 (5)*	2 (5)*	7 (2)	3 (2)*	4 (5)*	-1 (3)*	-1 (5)*	4 (4)*
YVEANMGLNPSSPNDPVTNICQ	3	249	270	H2, H3	1 (5)*	1 (4)*	0 (2)*	-2 (6)*	2 (5)*	2 (5)*	4 (2)*	3 (6)*	2 (4)*	2 (5)*	0 (5)*	3 (4)*	-3 (2)*	-1 (6)*	-2 (4)*	-2 (2)*	4 (4)*	5 (4)	4 (3)*	1 (6)*	1 (5)*	0 (2)*	2 (4)*	1 (2)*	1 (5)*	-1 (5)*	-1 (4)*	1 (3)*
ANMGLNPSSPNDPVTNICQ	2	252	270		3 (4)*	1 (5)*	0 (2)*	-3 (6)*	3 (5)*	2 (6)*	0 (5)*	-1 (6)*	-1 (5)*	-2 (5)*	0 (3)*	2 (4)*	-2 (4)*	-2 (5)*	-1 (4)*	-2 (2)*	2 (4)*	1 (7)*	2 (4)*	1 (5)*	-1 (5)*	0 (2)*	5 (4)	4 (3)*	1 (5)*	-2 (5)*	-1 (5)*	2 (2)*
AADKQLFTL	2	271	279	H3	-2 (5)*	0 (5)*	-2 (5)*	-11 (4)	2 (4)*	2 (5)*	1 (4)*	0 (5)*	0 (4)*	-1 (4)*	3 (5)*	0 (4)*	-7 (4)	-6 (5)	-6 (4)	-7 (4)	0 (4)*	2 (6)*	2 (5)*	2 (3)*	-1 (4)*	0 (5)*	-2 (6)*	0 (4)*	-14 (5)	-2 (5)*	-1 (5)*	0 (4)*
AADKQL	2	271	276		-2 (5)*	1 (5)*	-1 (5)*	-12 (5)	2 (6)*	1 (6)*	-2 (5)*	-3 (6)*	-3 (5)*	-4 (5)*	1 (3)*	-1 (3)*	-7 (5)	-5 (2)	-5 (4)	-6 (5)	0 (5)*	2 (7)*	3 (5)*	2 (2)*	0 (3)*	-4 (5)*	N/A	-1 (5)*	-12 (5)	-1 (5)*	0 (2)*	0 (4)*
LVEWAKRIPHF	3	279	289	H3, loop	0 (2)*	0 (3)*	0 (1)*	0 (1)*	0 (1)*	0 (1)*	0 (5)*	0 (4)*	-2 (3)*	-1 (1)*	1 (2)*	0 (1)*	3 (4)*	2 (2)*	1 (2)*	0 (1)*	0 (1)*	0 (1)*	0 (1)*	0 (1)*	-1 (2)*	-1 (1)*	0 (1)*	0 (2)*	2 (3)*	0 (2)*	-1 (2)*	0 (3)*
VEWAKRIPHFSELPLDDQ	3	280	297	H3, H4	0 (1)*	0 (1)*	0 (1)*	0 (1)*	0 (1)*	0 (1)*	1 (1)*	1 (2)*	0 (1)*	0 (1)*	0 (3)*	0 (1)*	-2 (2)*	-1 (2)*	-1 (1)*	-1 (1)*	0 (2)*	0 (2)*	0 (1)*	-1 (2)*	0 (1)*	0 (2)*	1 (2)*	1 (4)*	2 (2)*	-1 (2)*	-1 (4)*	0 (2)*
WAKRIPHFSELPLDD	4	282	296		0 (2)*	1 (1)*	1 (1)*	-2 (2)*	0 (1)*	0 (2)*	N/A	1 (2)*	1 (1)*	1 (1)*	0 (0)*	-2 (1)*	-2 (2)*	-1 (2)*	-3 (2)*	-3 (1)*	0 (2)*	0 (3)*	0 (1)*	-1 (2)*	-3 (4)*	0 (3)*	1 (2)*	0 (2)*	2 (2)*	0 (2)*	-1 (5)*	0 (2)*
WAKRIPHFSELPLDDQ	3	282	297		0 (2)*	0 (1)*	0 (1)*	0 (1)*	0 (1)*	0 (2)*	2 (2)*	2 (2)*	2 (1)*	2 (1)*	0 (3)*	1 (2)*	-3 (2)*	-2 (2)*	2 (2)*	3 (5)*	0 (2)*	0 (3)*	0 (1)*	-1 (2)*	5 (2)*	1 (3)*	1 (3)*	5 (5)*	2 (2)*	-1 (2)*	-1 (5)*	4 (3)*
LRAGWNELL	2	301	309	H5	0 (2)*	0 (1)*	0 (1)*	0 (1)*	0 (1)*	0 (1)*	1 (1)*	0 (1)*	1 (1)*	0 (1)*	0 (2)*	0 (1)*	0 (1)*	0 (1)*	0 (1)*	0 (1)*	0 (2)*	0 (1)*	0 (1)*	0 (1)*	0 (1)*	0 (1)*	2 (1)*	0 (2)*	2 (5)*	0 (3)*	0 (2)*	0 (4)*
LRAGWNEL	2	301	308		-1 (2)*	0 (1)*	0 (1)*	0 (1)*	0 (1)*	0 (1)*	0 (2)*	0 (1)*	-1 (1)*	-1 (1)*	3 (2)*	1 (1)*	2 (2)*	1 (1)*	1 (1)*	1 (1)*	0 (2)*	0 (2)*	0 (1)*	0 (2)*	3 (2)*	0 (2)*	2 (2)*	0 (2)*	2 (5)*	0 (3)*	0 (2)*	0 (4)*
LIASFSHRSIAVKDGIL	2	309	325	H5, S1	0 (4)*	0 (2)*	4 (2)*	1 (2)*	1 (1)*	1 (2)*	0 (3)*	-1 (3)*	1 (2)*	1 (2)*	3 (4)*	-2 (2)*	-1 (3)*	0 (2)*	0 (2)*	0 (2)*	1 (3)*	0 (4)*	0 (2)*	0 (3)*	5 (2)*	3 (4)*	3 (2)*	2 (3)*	3 (3)*	0 (5)*	-2 (3)*	3 (3)*
LATGLHVHRNSAHSAGVGAIF	3	326	346	S2, H6, H7	0 (4)*	1 (3)*	0 (3)*	-3 (3)*	1 (3)*	1 (4)*	8 (4)	6 (5)	9 (3)	9 (3)	1 (3)*	-2 (3)*	-4 (4)*	-4 (4)*	-5 (4)*	-4 (2)*	2 (3)*	1 (5)*	1 (3)*	0 (4)*	0 (4)*	0 (3)*	3 (3)*	-1 (3)*	-2 (4)*	-1 (3)*	-3 (5)*	1 (4)*
HVHRNSAHSAGVGAIF	4	331	346		-1 (4)*	1 (3)*	0 (3)*	-3 (4)*	1 (3)*	1 (4)*	-1 (7)*	0 (5)*	1 (3)*	0 (4)*	0 (4)*	-1 (3)*	1 (6)*	-1 (4)*	-2 (3)*	-3 (4)*	3 (4)*	N/A	0 (3)*	-1 (4)*	3 (4)*	N/A	4 (3)*	-2 (3)*	-2 (3)*	-1 (3)*	-1 (4)*	1 (3)*
VSKMRDMQM	2	354	362	H7, loop	-7 (3)	-8 (2)	-7 (2)	-4 (2)*	-6 (2)	-5 (2)*	-3 (4)*	-2 (4)*	-4 (2)*	-4 (2)*	0 (3)*	-1 (2)*	0 (2)*	0 (2)*	0 (2)*	-1 (1)*	0 (4)*	-2 (3)*	-4 (3)*	-4 (2)*	1 (2)*	1 (2)*	0 (4)*	-1 (3)*	4 (4)*	0 (6)*	-3 (6)*	1 (4)*
DKTELGCL	2	363	370	H8	5 (3)*	2 (1)*	2 (1)*	1 (1)*	-3 (2)*	-3 (2)*	-2 (1)*	-1 (1)*	-1 (2)*	-1 (2)*	-1 (2)*	0 (1)*	-2 (1)*	-1 (1)*	1 (2)*	1 (1)*	-2 (2)*	-2 (2)*	0 (2)*	-2 (2)*	0 (3)*	2 (2)*	-1 (3)*	2 (3)*	1 (5)*	0 (2)*	0 (3)*	-1 (3)*
RAIVLFNPDSKGLSNPAEVE	3	371	390	H8, H9	-1 (4)*	2 (2)*	1 (2)*	-1 (2)*	-1 (2)*	-2 (2)*	-2 (2)*	-2 (2)*	-6 (2)	-3 (2)*	2 (4)*	-1 (2)*	N/A	-2 (3)*	-2 (3)*	-2 (2)*	0 (3)*	-1 (3)*	-1 (2)*	-2 (3)*	-2 (3)*	1 (4)*	0 (2)*	-1 (4)*	1 (4)*	-1 (4)*	-1 (5)*	0 (4)*
RAIVLFNPDSKGLSNPAEVEA	3	371	391		-2 (2)*	-2 (1)*	-2 (1)*	-1 (2)*	-1 (1)*	-1 (2)*	-1 (2)*	-1 (2)*	-1 (1)*	-1 (1)*	-1 (3)*	-4 (2)*	4 (4)*	4 (4)*	-1 (4)*	0 (2)*	0 (2)*	-1 (3)*	-1 (1)*	-1 (3)*	0 (2)*	3 (3)*	1 (2)*	-2 (3)*	1 (4)*	-1 (3)*	-1 (5)*	-2 (3)*
IVLFNPDSKGLSNPAE	2	373	388		-2 (4)*	-2 (3)*	-2 (2)*	-1 (2)*	-2 (2)*	-2 (3)*	-4 (3)*	-4 (3)*	-4 (2)*	-4 (3)*	1 (5)*	0 (2)*	0 (3)*	0 (3)*	-1 (3)*	-1 (2)*	0 (3)*	-2 (4)*	-2 (3)*	-3 (4)*	1 (3)*	0 (4)*	2 (3)*	0 (4)*	1 (4)*	-2 (5)*	-2 (7)*	1 (5)*
IVLFNPDSKGLSNPAEVEA	2	373	391		-1 (3)*	-1 (2)*	-1 (2)*	-1 (2)*	-1 (2)*	-2 (2)*	-2 (2)*	-2 (2)*	-3 (2)*	-3 (2)*	-3 (3)*	-4 (2)*	-3 (1)*	-4 (3)*	-3 (2)*	-3 (1)*	0 (3)*	-1 (3)*	-1 (2)*	-2 (3)*	-1 (2)*	-1 (1)*	-1 (2)*	-2 (4)*	2 (4)*	-2 (4)*	-2 (5)*	-2 (1)*
IVLFNPDSKGLSNPAEVE	2	373	390		-2 (3)*	-1 (2)*	-1 (2)*	-1 (2)*	-2 (2)*	-2 (2)*	-3 (3)*	-2 (2)*	-3 (2)*	-4 (2)*	1 (4)*	-1 (2)*	-2 (2)*	-1 (2)*	-2 (2)*	-2 (1)*	0 (3)*	-1 (3)*	-2 (2)*	-2 (4)*	-2 (2)*	-1 (3)*	-2 (3)*	-1 (4)*	2 (4)*	-2 (4)*	-2 (6)*	-1 (4)*
FNPDSKGLSNPAEVE	2	376	390		-2 (5)*	-2 (3)*	-2 (2)*	-2 (3)*	-3 (4)*	-3 (3)*	-6 (3)	-7 (3)	-9 (3)	-9 (3)	1 (3)*	0 (3)*	2 (2)*	-2 (3)*	-2 (3)*	0 (2)*	1 (4)*	-1 (5)*	-2 (3)*	-3 (5)*	-1 (3)*	-1 (4)*	0 (4)*	-2 (6)*	2 (5)*	-2 (6)*	-3 (4)*	0 (5)*
FNPDSKGLSNPAEVEA	2	376	391		-3 (4)*	-2 (3)*	-3 (3)*	-1 (3)*	-1 (3)*	-3 (3)*	-5 (4)	-5 (4)*	-5 (3)*	-5 (3)*	1 (5)*	2 (3)*	1 (3)*	2 (3)*	5 (3)*	-2 (3)*	0 (4)*	-1 (4)*	-2 (4)*	-3 (4)*	-3 (4)*	-2 (3)*	-2 (3)*	-2 (5)*	2 (4)*	-2 (5)*	-2 (5)*	-1 (5)*
FNPDSKGLSNPAE	2	376	388		N/A	-2 (4)*	-3 (3)*	-3 (4)*	-3 (4)*	-4 (5)*	-7 (5)	-7 (4)	-7 (4)	-7 (4)	3 (7)*	0 (4)*	0 (4)*	2 (4)*	-1 (4)*	-1 (2)*	1 (5)*	-2 (5)*	-3 (4)*	-3 (5)*	4 (4)*	2 (5)*	1 (3)*	2 (5)*	1 (5)*	0 (5)*	-3 (4)*	3 (2)*
ALREKVYASL	2	391	400	H8, H9	0 (1)*	0 (0)*	0 (0)*	0 (1)*	0 (0)*	0 (0)*	1 (1)*	1 (1)*	1 (1)*	1 (1)*	0 (0)*	-1 (1)*	-1 (1)*	-1 (1)*	-1 (0)*	-1 (0)*	0 (0)*	0 (1)*	0 (1)*	0 (0)*	0 (1)*	-1 (0)*	0 (4)*	-1 (1)*	1 (1)*	0 (1)*	0 (0)*	-1 (2)*
LREKVYASL	2	392	400		0 (1)*	0 (0)*	0 (0)*	0 (1)*	0 (0)*	0 (1)*	N/A	1 (1)*	1 (0)*	1 (1)*	0 (0)*	-1 (0)*	-2 (1)*	-1 (1)*	-1 (0)*	-1 (0)*	0 (1)*	0 (1)*	0 (0)*	0 (0)*	-1 (1)*	-2 (0)*	-1 (1)*	0 (1)*	1 (1)*	0 (1)*	0 (0)*	-1 (3)*
EAYCKHKYPEQPGRF	3	401	415	H9, loop	0 (3)*	0 (2)*	0 (2)*	0 (3)*	0 (2)*	-1 (2)*	-3 (3)*	-4 (3)*	-4 (2)*	-4 (2)*	1 (4)*	0 (2)*	0 (3)*	0 (3)*	0 (2)*	0 (2)*	1 (3)*	-1 (4)*	0 (3)*	-1 (3)*	1 (3)*	1 (2)*	3 (2)*	0 (2)*	1 (2)*	-1 (4)*	-2 (4)*	1 (4)*
YCKHKYPEQPGRF	3	403	415		0 (3)*	0 (2)*	0 (2)*	-2 (3)*	0 (2)*	-1 (2)*	-5 (5)*	-5 (3)	-6 (3)	-6 (4)	2 (5)*	0 (3)*	2 (4)*	0 (3)*	1 (3)*	0 (4)*	2 (3)*	-2 (2)*	0 (5)*	-2 (4)*	1 (4)*	6 (3)	4 (6)*	-2 (1)*	1 (3)*	-1 (4)*	-2 (5)*	1 (4)*
LLRLPALRSIGL	3	419	430	H10	-10 (3)	-12 (2)	-12 (2)	-8 (3)	-14 (1)	-13 (2)	-10 (2)	-6 (2)	-11 (1)	-10 (1)	0 (1)*	-3 (2)*	-1 (1)*	0 (2)*	0 (1)*	-1 (1)*	-4 (1)	-6 (2)	-7 (2)	-7 (1)	0 (1)*	0 (0)*	-1 (1)*	-1 (1)*	0 (3)*	-1 (6)*	-2 (4)*	0 (3)*
LRLPALRSIGL	3	420	430		-11 (3)	-13 (3)	-13 (3)	-8 (3)	-16 (2)	-14 (2)	-12 (3)	-6 (4)	-11 (2)	-10 (2)	-1 (1)*	-4 (2)*	-1 (2)*	-1 (3)*	-2 (1)*	-2 (2)*	-4 (2)	-6 (2)	-8 (2)	-7 (2)	-1 (1)*	-1 (1)*	-1 (2)*	-2 (1)*	1 (3)*	-1 (5)*	-2 (5)*	-1 (5)*
PALRSIGL	2	423	430		-12 (3)	-14 (3)	-14 (2)	-8 (3)	-11 (1)	-12 (2)	-8 (2)	N/A	-9 (1)	-8 (1)	2 (2)*	-1 (2)*	3 (2)*	2 (2)*	2 (1)*	1 (1)*	-3 (1)*	-4 (2)*	-6 (2)	-4 (2)*	4 (2)*	4 (2)*	3 (2)*	0 (2)*	0 (5)*	-1 (5)*	-3 (4)*	1 (5)*
LEHLFF	2	433	438	H10, H11	-1 (4)*	1 (4)*	1 (4)*	-9 (5)	2 (4)*	1 (5)*	4 (4)*	3 (5)*	4 (4)*	4 (4)*	0 (2)*	-1 (4)*	-5 (3)*	-4 (5)*	-5 (3)	-5 (2)*	2 (4)*	1 (6)*	2 (4)*	-1 (4)*	-3 (4)*	-2 (3)*	0 (4)*	1 (3)*	-15 (4)	-3 (4)*	0 (4)*	-1 (4)*
FFKLIGDTPIDT	2	438	449	H11, H12	0 (3)*	1 (4)*	0 (4)*	-8 (4)	2 (4)*	1 (5)*	2 (4)*	1 (5)*	3 (4)*	3 (4)*	2 (3)*	-3 (5)*	-6 (3)	-5 (5)	-7 (4)	-7 (5)	2 (3)*	1 (5)*	1 (5)*	0 (3)*	-2 (3)*	-3 (5)*	-1 (4)*	-1 (4)*	-8 (3)	-2 (5)*	-2 (3)*	-1 (5)*
FKLIGDTPID	2	439	448		-1 (3)*	1 (4)*	0 (4)*	-9 (5)	0 (3)*	0 (6)*	-1 (5)*	-2 (6)*	-1 (4)*	-1 (5)*	0 (3)*	-1 (3)*	-7 (4)	-7 (4)	-7 (4)	-8 (5)	2 (5)*	0 (5)*	1 (6)*	0 (3)*	-4 (3)*	-1 (4)*	-3 (5)*	-1 (4)*	-9 (4)	-1 (4)*	-2 (2)*	-1 (4)*
FKLIGDTPIDTF	2	439	450		1 (4)*	1 (4)*	0 (4)*	-8 (4)	2 (4)*	1 (5)*	2 (4)*	1 (5)*	3 (4)*	3 (4)*	0 (3)*	-3 (5)*	-6 (3)	-3 (3)	-7 (4)	-7 (5)	2 (4)*	1 (5)*	0 (3)*	-2 (9)*	-2 (5)*	-3 (5)*	-1 (4)*	-1 (4)*	-8 (3)	-2 (5)*	4 (3)*	-1 (5)*
MEMLEAPHQMT	2	452	462	H12	-1 (5)*	1 (6)*	1 (4)*	-1 (3)*	1 (4)*	1 (4)*	-1 (4)*	-2 (5)*	-6 (4)	-7 (4)	0 (0)*	1 (4)*	-1 (3)*	-4 (3)	-2 (4)*	0 (3)*	2 (3)*	-2 (6)*	3 (4)*	-2 (5)*	1 (3)*	2 (4)*	1 (4)*	-1 (5)*	1 (4)*	-1 (5)*	0 (5)*	1 (2)*
MLEAPHQMT	2	454	462		-1 (2)*	1 (5)*	1 (5)*	-1 (5)*	1 (5)*	0 (5)*	-3 (5)*	-3 (5)*	-6 (5)	-6 (5)	0 (2)*	0 (5)*	-2 (4)*	-2 (5)	-4 (2)	-5 (2)*	2 (5)*	0 (5)*	0 (4)*	0 (6)*	0 (4)*	2 (5)*	2 (3)*	0 (5)*	3 (5)*	0 (5)*	-1 (5)*	0 (4)*
LEAPHQMT	2	455	462		0 (5)*	1 (4)*	1 (4)*	-1 (5)*	1 (4)*	0 (5)*	3 (5)*	2 (7)*	-2 (4)*	-2 (4)*	1 (5)*	0 (4)*	-6 (5)	-5 (4)	-6 (3)	-6 (3)	2 (5)*	1 (2)*	1 (4)*	-2 (6)*	-1 (4)*	1 (5)*	1 (5)*	1 (5)*	2 (4)*	-1 (5)*	-1 (4)*	1 (5)*

(xvii*)	(xix*)	(xxi*)	(xxiii*)
2 (5)*	1 (5)*	-1 (4)*	3 (3)*
2 (2)*	1 (4)*	-3 (5)*	2 (3)*
3 (3)*	2 (5)*	-3 (6)*	2 (5)*
1 (3)*	1 (4)*	-2 (5)*	1 (3)*
1 (3)*	2 (5)*	2 (4)*	1 (4)*
1 (4)*	2 (5)*	3 (3)*	3 (3)*
2 (3)*	1 (5)*	2 (3)*	-1 (4)*
2 (4)*	2 (5)*	-1 (5)*	2 (3)*
1 (3)*	1 (4)*	-1 (4)*	1 (2)*
0 (5)*	1 (5)*	0 (4)*	-3 (2)*
0 (4)*	0 (4)*	2 (3)*	-2 (5)*
-11 (4)	-11 (2)	-3 (3)*	-1 (4)*
-11 (3)	-10 (4)	-4 (3)*	-2 (0)*
-7 (2)	-8 (3)	-4 (2)*	-2 (3)*
-5 (3)	0 (4)*	1 (3)*	-4 (2)*
-8 (5)	-11 (5)	-2 (4)*	1 (3)*
-4 (2)*	-2 (3)*	0 (1)*	0 (1)*
1 (5)*	-2 (1)*	1 (5)*	1 (3)*
0 (3)*	0 (6)*	0 (5)*	2 (4)*
1 (4)*	0 (5)*	-2 (5)*	0 (1)*
2 (3)*	1 (3)*	-1 (4)*	2 (3)*
3 (4)*	3 (5)*	-2 (4)*	2 (4)*
1 (4)*	4 (3)*	-2 (3)*	2 (5)*
0 (4)*	4 (4)*	0 (5)*	0 (5)*
1 (2)*	0 (3)*	1 (2)*	-1 (2)*
1 (3)*	2 (0)*	-2 (2)*	0 (3)*
2 (3)*	1 (4)*	-2 (2)*	-1 (2)*
2 (3)*	1 (4)*	3 (2)*	-2 (5)*
1 (2)*	0 (2)*	0 (2)*	-2 (1)*
0 (2)*	0 (2)*	0 (2)*	2 (2)*
3 (4)*	1 (4)*	3 (3)*	-2 (2)*
2 (4)*	2 (3)*	-5 (2)*	3 (3)*
3 (5)*	0 (3)*	1 (5)*	0 (2)*
0 (4)*	-2 (3)*	-2 (2)*	1 (2)*
-2 (2)*	-1 (3)*	-1 (2)*	4 (5)*
2 (4)*	-1 (3)*	2 (3)*	2 (2)*
2 (4)*	1 (2)*	2 (2)*	-2 (2)*
2 (5)*	-1 (3)*	-3 (4)*	0 (3)*
2 (4)*	-1 (3)*	-3 (3)*	-4 (3)*
2 (4)*	-1 (3)*	-3 (3)*	-3 (2)*
3 (6)*	-1 (4)*	-3 (2)*	-1 (4)*
2 (6)*	-1 (4)*	-1 (4)*	-4 (3)*
0 (0)*	1 (2)*	-1 (0)*	2 (3)*
0 (0)*	1 (5)*	-1 (0)*	-1 (3)*
2 (4)*	-1 (4)*	-2 (3)*	0 (2)*
4 (4)*	-1 (3)*	-3 (2)*	0 (4)*
-4 (1)	-6 (3)	-1 (1)*	-1 (2)*
-4 (2)	-3 (2)*	-1 (1)*	-1 (1)*
-2 (2)*	-3 (1)*	3 (2)*	2 (3)*
2 (4)*	5 (4)*	-2 (2)*	-2 (3)*
2 (4)*	3 (4)*	-3 (1)*	-2 (3)*
1 (6)*	3 (5)*	-4 (2)*	0 (4)*
2 (4)*	3 (4)*	-3 (1)*	-2 (3)*
1 (4)*	2 (4)*	-1 (4)*	0 (1)*
1 (4)*	1 (5)*	-1 (4)*	2 (3)*
2 (4)*	-1 (5)*	-2 (4)*	1 (3)*

-50.0 -47.5 -45.0 -42.5 -40.0 -37.5 -35.0 -32.5 -30.0 -27.5 -25.0 -22.5 -20.0 -17.5 -15.0 -12.5 -10.0 -7.5 -5.0 -2.5 0.0 2.5 5.0 7.5 10.0 12.5 15.0 17.5 20.0 22.5 25.0 27.5 30.0 32.5 35.0 37.5 40.0 42.5 45.0 47.5 50.0

%D

Supplementary Figure 1d

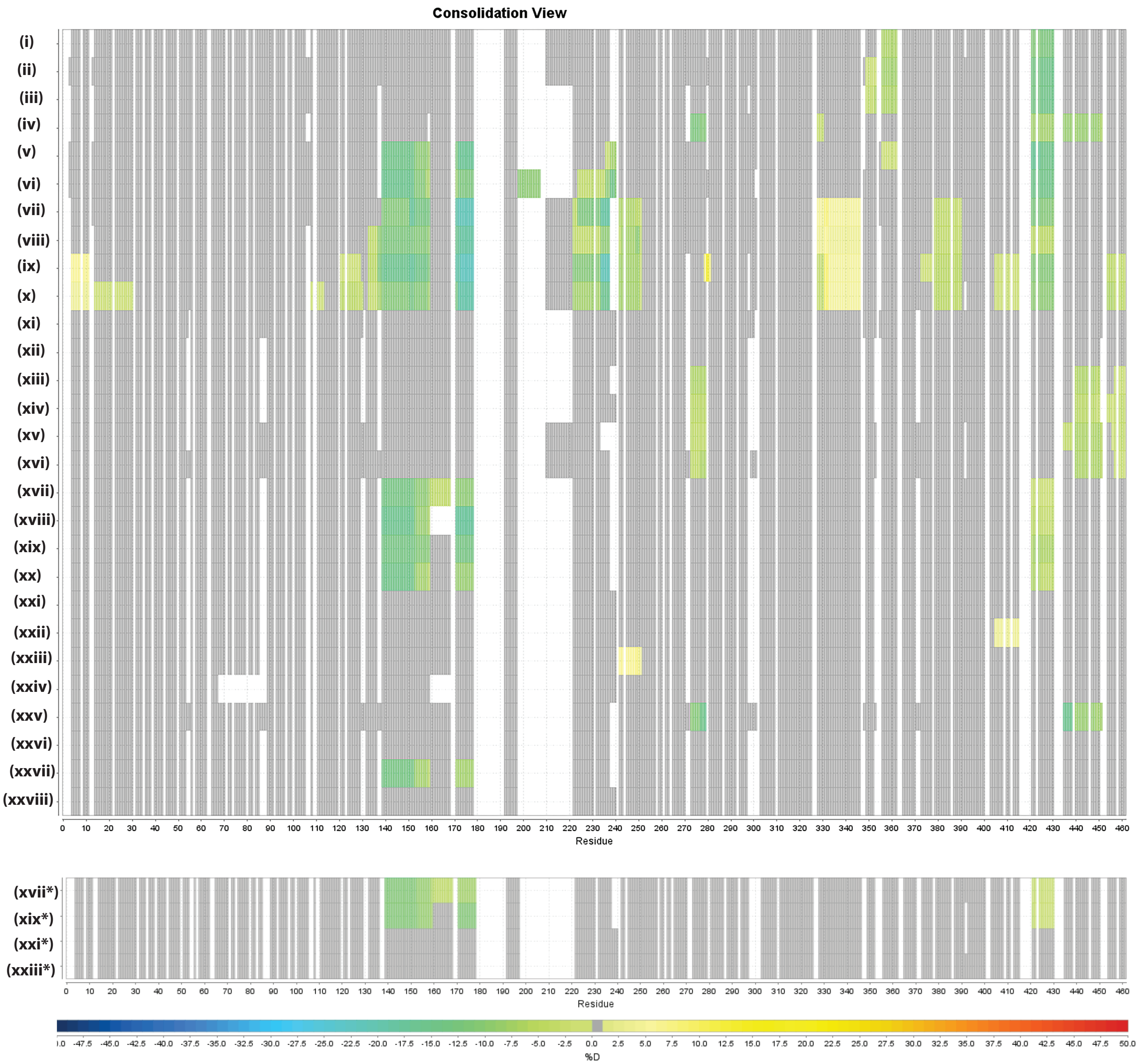

Supplementary Figure 1e

Supplementary Figure 1

A summary of all differential HDX experiments. (**a**) Schematic representations of all pairwise HDX experiments performed in this study. (**b**) Experimental comparison view for VDR subunit with the experimental order in **Supplementary Fig 1a**. (**c**) Experimental consolidation view for VDR subunit with the experimental order in **Supplementary Fig 1a**. (**d**) Experimental comparison view for RXRα subunit with the experimental order in **Supplementary Fig 1a**. (**e**) Experimental consolidation view for RXRα subunit with the experimental order in **Supplementary Fig 1a**.

The values listed under each HDX experiments demonstrate the averaged difference in percentage of deuterium incorporation of that corresponding peptide between two different states across all exchange time points (i.e., 10s, 30s, 60s, 300s, 900s, and 3600s). The regions with statistically significant differential deuterium incorporations were colored according to the smooth color gradient at the bottom. Statistical summary was from a two-way ANOVA between each pairwise experiment, $p<0.001$. A negative value represents decreased deuterium incorporation or stabilization while a positive value represents increased deuterium incorporation or de-stabilization in the corresponding region of the receptor when a binding event takes place. Peptides exhibiting statistically insignificant or undetectable changes are colored gray. N/A represents undetected peptide for corresponding experiment. The deuterium incorporation data from all overlapping peptides were consolidated to individual amino acid values using a residue averaging approach [1]. HDX Workbench was used to automate mapping of gradient colors to pymol structure model.

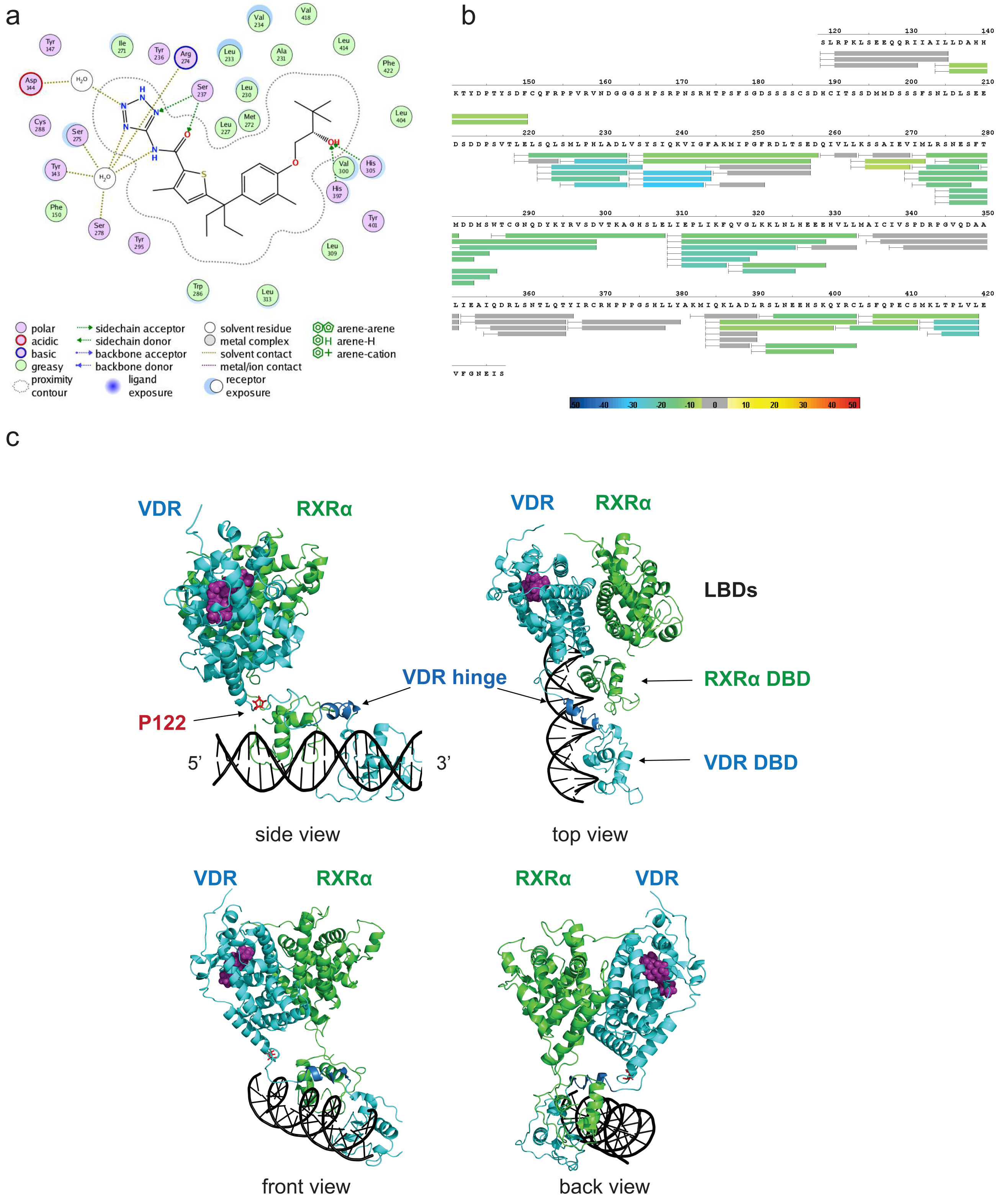

Supplementary Figure 2

d

VDR peptide: KRKEEEALKDSLRPKLSEEQQRIIA (+4)

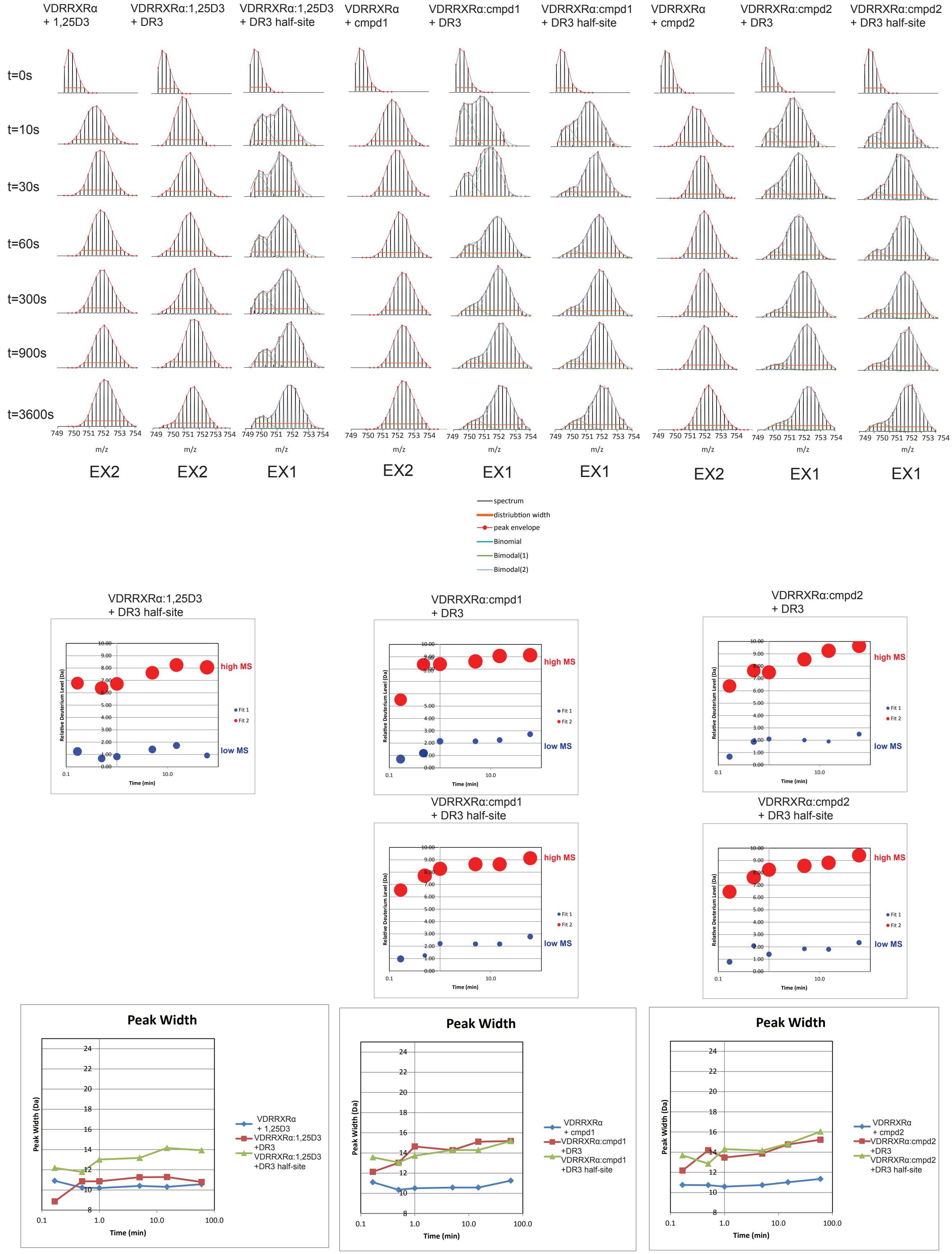

e

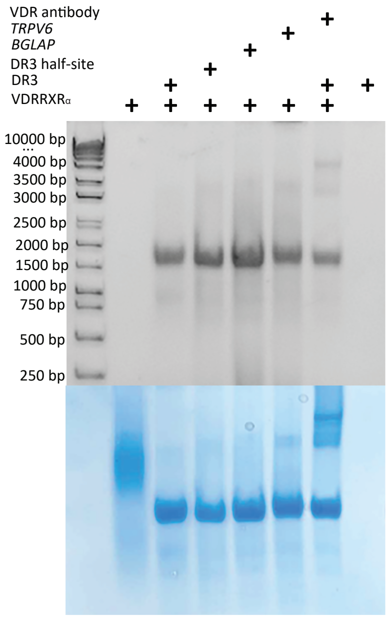

Supplementary Figure 2

Supplementary Figure 2. (a) Structural features of VDR LBD-ligand interaction (b) HDX analysis of VDR LBD upon binding of Cmpd3 VDRM. (c) Homology model* of VDRRXRα heterodimer DR3 complex based on CryoEM structure. Proline122 is shown in stick and color-coded in red; VDR, cyan; RXRα, green; 1,25 D3, purple; DR3, black. (d) EX1/EX2 kinetics of VDR hinge-H1 region. VDR hinge-H1 peptide (KRKEEEALKDSLRPKLSEEQQRIIA, +4) displays EX1 and EX2 kinetics upon heterodimer binding to different ligand and DNA. EX1 kinetics presents two distinct mass isotopic distributions with prolonged peak width whereas EX2 kinetics presents only one isotopic distribution. Upper panel: mass spectra of VDR hinge-H1 peptide derived from various complexes in indicated on-exchange time points. The isotopic distributions of MS peaks are analyzed by HX express 2 software[2,3]. Middle panel: MS spectra undergoing EX1 kinetics are characterized by fitting the low and high MS envelop, color-coded by blue and red bubble plot respectively. The bubble plot shows the exchange profiles with the relative size of each point reflecting the relative population of each species. Lower panel: peak width analysis by HX express2. Peak width was determined at 12% of peak intensity (e) EMSA analysis of VDRRXRα heterodimer VBS interactions. Native DNA gel retardation analysis of VDRRXRα heterodimer with various VBSs. *Apo* VDRRXRα heterodimer was incubated with or without indicated VBS – DR3, DR3 half-site, *BGLAP*, and *TRPV6* – to form different protein complex before native gel shift assays. Super-shift of DNA and protein was observed by further incubation of VDR antibody with heterodimer DNA complex.

* VDRRXRα DR3 model: the VDRRXRα heterodimer was created in the "open" conformation and a partial cryo-EM model (generous gift from Professor Dino Moras) was used to guide the creation of the model. VDRRXRα heterodimer forms an extended, L-shaped organization with RXRα DBD occupying one half-site of DR3 at 5'- end and VDR DBD occupying another at 3'-end [4,5]. VDR hinge domain, as a connection liker between DBD and LBD, plays a key role in determining structural orientation of LBD dimer, which is arranged perpendicular to DR3. Residue Proline122, which resides on the VDR hinge domain, was described as the "kink residue" between the C-terminal hinge helix and helix H1 of the LBD playing an important role in dictating the orientation of the LBD, resulting in an open LBD dimer architecture facing away from DBD. In this model, VDR hinge domain adopts an α-helical structure residing closely to DR3 and makes extensive contacts with phosphate and backbone of DNA[5]. This is in consisitency with our HDX observations that VDR hinge undergoes different extent of protections upon binding to various VBSs. Unlike VDR, DNA binding does not perturb RXRα

hinge dynamics as it forms a flexible linker that allows enhanced adaptability of heterodimer to diverse response elements. This model suggests that the hinges could possess intrinsic properties to orient LBD dimer in a precise way and provide structural basis for crosstalk between LBDs and DBDs.

Reference:

1. Keppel, T.R. & Weis, D.D. Mapping Residual Structure in Intrinsically Disordered Proteins at Residue Resolution Using Millisecond Hydrogen/Deuterium Exchange and Residue Averaging. *Journal of the American Society for Mass Spectrometry* **26**, 547-554 (2015).
2. Guttman, M., Weis, D.D., Engen, J.R. & Lee, K.K. Analysis of overlapped and noisy hydrogen/deuterium exchange mass spectra. *J Am Soc Mass Spectrom* **24**, 1906-12 (2013).
3. Weis, D.D., Wales, T.E., Engen, J.R., Hotchko, M. & Ten Eyck, L.F. Identification and characterization of EX1 kinetics in H/D exchange mass spectrometry by peak width analysis. *J Am Soc Mass Spectrom* **17**, 1498-509 (2006).
4. Nwachukwu, J.C. & Nettles, K.W. The nuclear receptor signalling scaffold: insights from full-length structures. *EMBO J* **31**, 251-3 (2012).
5. Orlov, I., Rochel, N., Moras, D. & Klaholz, B.P. Structure of the full human RXR/VDR nuclear receptor heterodimer complex with its DR3 target DNA. *EMBO J* **31**, 291-300 (2012).
