# Supplementary material for: HDX reveals the conformational dynamics of DNA sequence specific VDR co-activator interactions
Source: Nat Commun. 2017 Oct 13;8:923. doi: 10.1038/s41467-017-00978-7 (PMC5640644; doi:10.1038/s41467-017-00978-7)
Supplement: Supplementary file 3 — Description of Additional Supplementary Files [file 41467_2017_978_MOESM3_ESM.pdf]

Description of Supplementary Files

File name: Supplementary Data 1
Description: Chemical names, 1H NMR data, 13C NMR data, and high resolution mass spectrometry data for Compounds 1-3.
