# Supplementary material for: HDX reveals the conformational dynamics of DNA sequence specific VDR co-activator interactions
Source: Nat Commun. 2017 Oct 13;8:923. doi: 10.1038/s41467-017-00978-7 (PMC5640644; doi:10.1038/s41467-017-00978-7)
Supplement: Supplementary file 4 — Supplementary Data 1 [file 41467_2017_978_MOESM4_ESM.docx]

**Supplementary Data 1**

**Chemicals:**

Compound 1: [(4-{1-ethyl-1-[4-(2-hydroxy-3,3-dimethyl-butoxy)-3-methyl-phenyl]-propyl}-2-methyl-benzoyl)-methyl-amino]-acetic acid

1H NMR (400MHz, CDCl_3_), δ ppm: 0.56-0.63 (m, 6H), 1.02 (s, 9H), 2.01- 2.09 (m, 4H), 2.11 (s, 0.7H), 2.18 (s, 2.3H), 2.23 (s, 0.70H), 2.29 (s, 2.30H), 2.91 (s, 2.30H), 3.14 (s, 0.70H), 3.71 (dd, J = 8.8, 2.6 Hz, IH), 3.86 (t, J = 8.8 Hz, 1H), 3.92(s, 0.47H), 4.09 (dd, J = 8.8, 2.6 Hz, 1H), 4.33 (bs, 1.53H), 6.69 (d, J = 8.8 Hz, 0.23H), 6.70 15 (d, J = 8.3 Hz, 0.77H), 6.85-.11 (m, 5H).

13C NMR (400MHz, CDCl_3_), δ ppm: 8.37, 16.59, 19.15, 26.04, 29.03, 29.11, 33.56, 37.76, 48.64, 49.07, 69.10, 69.16, 110.15, 125.31, 125.66, 125.69, 126.20, 130.07, 130.65, 132.10, 133.79, 140.39, 150.30, 150.36, 154.46, 172.78, 173.15

HR-MS: 483.2979

Compound 2: L-2-[(5-{1-Ethyl-1-[4-(2-hydroxy-3,3-dimethyl-butoxy)-3-methyl-phenyl]-propyl}-3-ethyl-thiophene-2-carbonyl)-amino]-propionic acid.

1H NMR (400MHz, CDCl_3_), δ ppm: 0.71 (t, J= 7.4 Hz, 6H), 1.02 (s, 9H), 1.51 (d, J= 7.7 Hz, 3H), 2.04-2.14 (m, 4H), 2.20 (s, 3H), 2.47 (s, 3H), 3.72 (dd, J= 8.7, 2.5 Hz, 1H), 3.87 (t, J= 8.7 Hz, 1H), 4.10 (dd, J= 9.3,2.8 Hz, 1H), 4.64-4.72 (m, 1H), 6.22 (d, J = 7.4 Hz, 1H), 6.62 (s, 1H), 6.73 (d, J= 8.4 Hz, 1H), 6.97-7.06 (m, 2H).

13C NMR (400MHz, CDCl_3_), δ ppm: 8.34, 16.09, 16.64, 18.03, 26.04, 30.45, 33.58, 48.29, 48.75, 69.19, 77.20, 110.29, 125.52, 126.03, 127.03, 129.75, 129.96, 138.85, 142.06, 155.00, 157.94, 163.51, 176.12

HR-MS: 489.2538

Compound 3: 3'-[4-(2-hydroxy-3,3-dimethylbutoxy)-3-methylphenyl]-3'-[5-(tetrazol-5-yl-aminocarbonyl)- 4-methylthiophen-2-yl]pentane.

1HNMR (400MHz, DMSO-d6) δ ppm: 0.67 (t, J = 7.3 Hz, 6H), 0.93 (s, 9H), 2.00-2.15 (m, 4H), 2.13 (s, 3H), 2.46 (s, 3H), 3.46 (m, 1H), 3.77 (dd, J = 7.3,9.9 Hz, 1H), 4.04 (dd, J = 2.9, 10.2 Hz, 1H), 4.80 (d, J = 5.5 Hz, 1H), 6.87 (m, 2H), 7.04 (m, 2H), 11.80 (s, 1H), 15.92 (br s, 1H).

13C NMR (400MHz, CDCl_3_), δ ppm: 8.28, 16.03, 16.50, 26.09, 29.68, 33.98, 47.80, 69.94, 75.93, 110.55, 125.27, 125.36, 125.57, 129.12, 129.36, 137.63, 144.07, 150.31, 155.21, 159.38, 161.04

HR-MS: 485.2446
